# Support from parents, teachers, and peers and the moderation of subjective and objective stress of secondary school student

**DOI:** 10.1038/s41598-024-51802-4

**Published:** 2024-01-12

**Authors:** Frances Hoferichter, Jonne Lohilahti, Miriam Hufenbach, Hans Jörgen Grabe, Geja Hageman, Diana Raufelder

**Affiliations:** 1 Institute of Educational Science, University of Greifswald, Greifswald, Germany; 2 Institute of Psychology Greifswald, University of Greifswald, Greifswald, Germany; 3 Department of Psychiatry and Psychotherapy, University Medicine Greifswald, Greifswald, Germany; 4 Department of Pharmacology and Toxicology, Research Institute of Nutrition and Translational Research in Metabolism (NUTRIM), Maastricht University, Maastricht, The Netherlands

**Keywords:** Psychology, Biomarkers

## Abstract

During adolescence, students increasingly report suffering from stress and school burnout, which poses a risk to students’ healthy development. However, social support may counteract perceived stress according to the Buffering Hypothesis and the Conservation of Resources Theory. In search of factors that would support healthy student development, studies have primarily focused on self-report data and neglected biophysiological processes. Addressing this research desideratum, this study examined whether perceived social support buffers the interplay of self-reported stress considering biophysiological markers (i.e., cortisol, alpha-amylase, oxidative stress, and telomere length). 83 secondary school students (*M*_*age*_ = 13.72, *SD* = 0.67; 48% girls) from Germany participated in a questionnaire study and biophysiological testing. Moderation analyses in R revealed that support from parents moderated the relationships between psychological stress as well as cynicism and inadequacy at school linked to alpha-amylase.

## Introduction

The period of adolescence can prove to be demanding for students, given that they encounter a range of transformations. These encompass biological and cognitive shifts arising from maturation, alterations in their social interactions with both peers and parents, and adjustments prompted by heightened academic demands and expectations from school^1–4^. Accordingly, secondary school students commonly experience increased levels of psychological stress and school burnout^5–7^. However, there have been few studies investigating whether the self-perceived stress and overload symptoms are also related to biophysiological stress markers (e.g., cortisol, alpha-amylase, oxidative stress, telomere length). Even though students’ self-reported information is central to unlocking their feelings and thoughts, it is also susceptible to systematic error due to desired response behavior^8^. By merging subjective and biophysiological stress metrics, it becomes possible to uncover intricate psychophysical mechanisms associated with stress. These insights could potentially form the foundation for interventions aimed at managing stress. While the exploration of biophysiological stress markers like cortisol and alpha-amylase has gained attention in the educational setting in recent times^9–11^, the examination of biophysiological indicators associated with stress and arousal, such as oxidative stress and telomere length, has predominantly been conducted in the realm of psychopathology and health studies. Nonetheless, delving into a wide range of biophysiological markers within the educational context alongside self-report data from school students offers encouraging avenues for valuable insights, supporting the healthy development of students by identifying factors that can have a minimizing effect on students’ stress and burnout.

Both the buffering hypothesis^12^ as well as the Conservation of Resources theory COR^13^ posit that supportive social relationships have the potential to mitigate sensations of stress and overwhelm, serving as crucial assets for effectively managing challenging situations. Although there are numerous findings confirming the buffering hypothesis and COR with regard to the protective effects of social support e.g.,^14–16^, to the best of our knowledge, there has been no study to date that has also examined this approach in the interplay of self-perceived *and* biophysiological stress and arousal. The present study was designed to address this research desideratum by examining how social relationships with parents, teachers, and peers that are perceived as supportive moderate the relationship between subjective stress (e.g., school burnout, perceived stress) and biophysiological stress markers (e.g., cortisol, alpha-amylase, oxidative stress, telomere length).

### Subjective stress, cortisol, alpha-amylase, oxidative stress, and telomere length

Psychological stress may arise if demands exceed an individual’s resources which is associated with increased threat appraisals and negative affect^17,18^. While psychological stress is not primarily related to a specific context, school burnout is experienced in relation to the academic context. Students often report school-related psychosomatic stress symptoms such as head-aches or dizziness if they feel pressured to receive high grades, worry about their academic performance, and feel overwhelmed by schoolwork^19,20^. Long-lasting and high levels of school-related psychosomatic stress symptoms make students more susceptible to school burnout, which includes exhaustion from schoolwork, feeling inadequate as a student, and adopting a cynical and detached attitude towards school^21,22^. Although psychological stress and school burnout are conceptualized and measured differently, the constructs are related^23^ and present a risk to students’ health and academic trajectories^24–29^.

Several large-scale international surveys have demonstrated steadily increasing levels of self-reported psychological stress, school-related psychosomatic stress symptoms, and school burnout both in the cohort of secondary school students and over the last decade^30–34^. Because of this alarming increase, it is also central to combine research approaches and ask whether or to what extent self-perceived stress and burnout are related to a variety of biophysiological stress markers.

### Cortisol and alpha-amylase

Cortisol belongs to the glucocorticoid class of hormones and is commonly referred to as the “stress hormone,” while amylase is an enzyme that has been investigated as a suitable candidate to measure stress-related arousal^35,36^. While alpha-amylase has traditionally been studied in the context of stress, research outside the clinical context has revealed its involvement in both heightened arousal of positive emotions and negative emotions experienced by individuals^37^. Hence, while cortisol has been consistently linked to stress related arousal, alpha-amylase is not limited specifically to stress or negative emotional valence.

While both biological indicators serve as valuable markers for detecting physiological changes in response to arousal, cortisol is reflective of the hypothalamic–pituitary–adrenal (HPA) axis and α-amylase is a reliable index of sympathetic-adrenal medullary (SAM) activity during arousal^36,38^. As both reflect the reactivity of distinct bodily stress systems, some studies speak of an asymmetry between the HPA axis and SAM in a bodily response to arousal^39^ that is related to an asymmetry between cortisol and α-amylase reactivity^40^. For this reason, both act differently with respect to timely release in response to a stress-induced task^41^, they relate differently to self-reported stress^42,43^, and they may not be correlated according to the findings of some studies^10,36,44^.

There is a general belief that long-term stress leads to an increase in cortisol levels^45^. In this case, the overactivation of the HPA axis for a prolonged period of time induces wear and tear on the stress systems, which relates to negative health outcomes^46,47^. In this respect, Jamieson et al.^48^ demonstrated that both challenge and threat appraisals activate the HPA axis, while threat appraisals have a stronger activation potential than challenge. Lee and colleagues have described cortisol to be an end product of threat-type stress responses^49^. However, empirical studies on the relation between self-reported stress, cortisol and alpha-amylase levels among healthy individuals are rather complex.

### Oxidative stress

Oxidative stress introduces an additional dimension to the detection of biophysiological stress responses. It reflects an asymmetry between the production of reactive oxygen species (commonly known as free radicals) and the protective actions of antioxidants^50^. Numerous human and animal studies point to a correlation between psychosocial stress and elevated oxidative stress levels, which consequently contribute to cellular and tissue damage within the body^51–54^. Nonetheless, investigations into the connection among oxidative stress and subjective stress within the school context are so far uncommon.

### Telomere length

Telomeres consist of repetitive DNA sequences that provide stability to the ends of chromosomes, gradually shortening as a result of cell division over time^55^. Moreover, a link has been established between psychosocial stressors and the hastening of biological aging. This linkage is attributed to chronic stress directly influencing cellular mechanisms associated with the emergence of different diseases and the reduction of telomere length^56,57^. While telomeres have primarily been the focus of clinical research, a recent study conducted in an educational context revealed that school students with longer telomeres were less likely to report experiencing burnout^58^.

In sum, empirical findings—mainly in the field of psychopathology and health studies—have indicated that cortisol, α-amylase, oxidative stress, and the length of telomeres are reliable but distinct indicators of biological stress and arousal. However, research that links these biophysiological markers with school students’ reported stress and burnout remains underdeveloped. Establishing connections between subjective and biophysiological stress linked to social support variables offers a promising strategy for preventing students from encountering psychological stress and school-related burnout, thereby promoting healthy psychological and academic development. Therefore, it is essential to identify resources that are accessible within students’ immediate environment and protect them from experiencing strain, stress-related negative affect, and feelings of being overwhelmed by school. Research has demonstrated that nurturing relationships with parents, teachers, and peers constitute significant resources that can mitigate feelings of stress in school students.

### Social support and students’ stress

Social support emerges as a potent and enduring coping strategy during challenging circumstances, recognized as a fundamental cornerstone of social, psychological, and biological well-being^59^. Social buffering, which falls within the realm of social support, refers to the mechanism by which the presence of a similar species diminishes the activity of stress-related neurobiological systems^60^. In a human context, Cohen and Wills^12^ introduced the buffering hypothesis, which proposes that relationships perceived as supportive serve to alleviate the impact of stress. Likewise, the Conservation of Resources Theory COR^13^ posits that individuals endeavor to preserve their resources, including their capacity for well-being, which necessitates the utilization of resources such as supportive social relationships^59^. Hence, feelings of psychological stress and school burnout draw on students’ resources and consequently prompt a need for more resources, such as supportive social relationships, to help regain and maintain well-being. Many empirical studies have investigated students’ stress levels and well-being—as a counterweight to stress—in relation to their supportive relationships with parents, teachers, and peers, but their results differ depending on the measures used to capture social support, the age group investigated, and the statistical models tested.

For example, studies have found that parental support protects students from feeling overwhelmed by school and is related to low levels of student’s stress^14,61^. Parental support can buffer the impact of stress on adolescents and facilitate school enjoyment^62,63^. Hostinar et al.^64^ found that social support from parents was related to lower cortisol levels in elementary school students compared to support from a stranger after students participated in the Trier Social Stress Test (TSST) the same did not apply to secondary school students. Raphael and Paul^65^ demonstrated that parental support measured by parent–child discussions about the future, joint school activities and parental school involvement were related to low levels of stress among secondary school students, but only in the absence of parental psychological control. The time parents spend with their children has been linked positively to children’s well-being^66,67^ and contributes to a lesser extend of problem behavior in adolescents^68^.

Besides support from parents, support from teachers has been shown to minimize the effect of stress on externalizing problems^69^ and contributes to students’ self-reported health related to stress^70,71^. A study with Chinese adolescents could show that teacher support improves students’ mental well-being and resilience^72^. Another study with German adolescents could show that perceived teacher support was positively related to students’ self-worth and physical well-being^15^. Empirical findings from the school context suggest that students’ stress levels are buffered by perceived support from teachers^29,73,74^.

In addition, support from peers can minimize stress symptoms. Adams et al.^75^ detected that the presence of a best friend was related to low levels of cortisol during the exposure to TSST in elementary students. Investigating sAA and the reported network size of students, Ponzi et al.^76^ found that the ego reported network size was correlated with higher sAA levels, indicated higher physiological arousal of students with larger network sizes.

In quantitative questionnaire studies, the link between social support by peers—also measured by classroom climate—and students’ stress has been demonstrated consistently. For instance, Torsheim and Wold^74^ revealed that support from peers was related to lower levels of school-related stress, a finding that was also confirmed by Hoferichter et al.^77^. Similarly, Hoferichter and colleagues^78^ demonstrated that from grades seven to nine, classmate support buffered the increase of anti-school attitudes, which was related to school-related stress. However, according to a health study conducted by the World Health Organization (WHO), between the ages of 11 and 15, perceived support from classmates declined in nearly all of the 43 countries and regions across Europe and North America that were included in the study. Consequently, alternative social support sources, e.g., parental and teacher support or friends outside school, may compensate for the decline of peer support within the classroom.

In sum, secondary school students’ supportive relationships with parents, teachers, and peers represent valuable resources that help students overcome stressful situations and maintain their well-being. However, research on whether social support buffers the relationship between various measures of self-perceived stress and biophysiological markers linked to stress and arousal is still missing. In response to this need, the present study aligns with the recommendations of Hanssen et al.^79^ who proposed that future research on psychosocial stressors among youth should explore various stressors and explore potential factors that may moderate them. Hence, the objective is to gain a deeper understanding of the attributes of stressors and associated symptoms and to identify factors that predict vulnerability and resilience among young individuals.

#### Hypotheses

Hypothesis 1: Following the COR theory and empirical evidence, it is expected that social support from parents, teachers and peers is associated with lower levels of students’ psychological stress and school burnout (i.e., (1) exhaustion and (2) cynicism & inadequacy).

Exploratory Research Question: Given the underdeveloped state of research regarding the connection between biophysiological stress and arousal markers (i.e., cortisol, alpha-amylase, oxidative stress, telomeres) and support from parents, teachers, and peers, we do not propose a hypothesis but instead adopt an exploratory approach. Thereby it is investigated if the social support variables moderate the relationships between student’s perceived stress and burnout with their levels of cortisol, alpha-amylase, oxidative stress, and telomere lengths.

## Method

### Participants and procedure

The current sample is part of a larger sample (*N* = 733) of students from schools in Northeastern Germany, who participated in a survey investigating many different aspects of school-related stress and support factors during regular class hours. Out of these students, 100 participants were randomly selected and invited for the collection of biomarkers. Written informed consent was given by the parents after full explanation of the study to them and their child, with 83% consenting for both the questionnaire study and biophysiological testing (*N* = 83, *M*_*age*_ = 13.72, SD = 0.67; 48% girls). The study was approved by the Ministry of Education and Child Day Care Mecklenburg-Pomerania, the data protection officer and the ethics committee of the university medical centre. All methods performed in accordance with relevant guidelines and regulations.

To determine the participants’ salivary α-Amylase (sAA) and cortisol levels, they were instructed to collect a saliva sample independently after waking up using salivary cuvettes supplied by a team member of the study during the prior medical examination. Instructions were given on how to collect the sample correctly, i.e., to collect it immediately after waking up, before eating or drinking anything or brushing their teeth as well as how to recognize that they had collected an appropriate amount of saliva using indicators on the container.

On the day of the measurements, the participants woke up on average at 8:00 AM (*SD* = 1.29 h) and visited the University of Greifswald to give a second set of saliva samples at 10:54 AM on average (*SD* = 1.06 h). Here the students also provided urine samples for oxidative stress analysis as well as saliva samples to analyse telomere length. The salivettes containing the sAA and cortisol samples were stored at − 20 °C, while the urine samples were stored on ice and frozen at − 80 °C before analysis. The telomere length saliva was kept at room temperature in Oragene·DNA (OG-500) collection kits. Sample collection at the university was conducted by trained staff.

## Measures

### Self-reported stress measures (predictors)

*Psychological Stress.* This variable is a subscale of the Perceived Stress Scale PSS^80^, capturing the subjective stress level of the responder experienced during the last month. It consists of six items such as ‘How often did you feel nervous or “stressed” during the last month?’ on a five-point Likert scale ranging from 1 (‘never’) to 5 (‘very often’) with α = 0.87.

*School burnout inventory* The School Burnout Inventory SBI^21^ is a self-report questionnaire that inquires for symptoms of burnout in students, and as such for signs of adverse psychological consequences of long-term stress. The SBI measures burnout as a continuous variable and does not serve as diagnostic tool, e.g. by providing cut-offs for individuals at risk. The SBI consists of 9 items which are grouped into the three subscales ‘Cynicism’ (3 Items, e.g. ‘I constantly ask myself if my schoolwork is good for anything at all’), ‘Exhaustion’ (4 Items, e.g. ‘I feel overburdened by my schoolwork’) and ‘Inadequacy’ (2 Items, e.g. ‘I often feel like my schoolwork isn’t good enough’). Participants are asked to rate their agreement with these statements on a six-point Likert Scale ranging from 1 (‘Do not agree at all’) to 6 (‘Agree completely’). Following the literature^81^, we used a two factor approach consisting of the factor 1. exhaustion α = 0.82 and 2., a combined factor of inadequacy and cynicism α = 0.73, called ‘inadequacy/cynicism’ this study.

### Biophysiological stress markers (outcomes)

Salivary Alpha-Amylase and Salivary Cortisol were analyzed from morning and noon samples of participants. Oxidative stress was analyzed from urine provided at noon. Telomeres were analyzed from saliva provided at noon. For further description on the biophysiological measures and processing, see appendix on “Analyzes of biophysiological stress markers”.

### Self-reported social support variables (moderators)

*Parental Support.* We used the Social Capital Instrument based on works of the Max Planck institute for Educational Research and the PISA survey instrument, described in Kunter et al.^82^. This questionnaire consists of five activities parents engage in together with their children such as playing (i.e., board games), doing household chores, or having dinner together and asks the participant to rate how often these take place on a four-point Likert scale from 1 (‘never’) to 4 (‘very often’) with *α* = 0.80.

*Teacher and peer support* The ‘Teacher Classmate Support Scale’ was used^83^ and includes the Teacher Support subscale (four statements, incl. ‘Our teachers treat us fairly’) with *α* = 0.57. The Peer Support subscale (four statements, incl. ‘Most of my fellow students are friendly and ready to help’) with α = 0.78. Both subscales are arranged on a five-point Likert scale from 1 (‘don’t agree at all’) to 5 (‘agree completely’).

### Control variables

*Gender* The biological sex was of interest in this study and was coded as 0 = female (*n* = 40) and 1 = male (*n* = 43).

*School type* School type was divided into two possible answers: 0 = lower-track school (*n* = 41) and 1 = higher-track school (*n* = 42). While higher-track schools provide students with a school leaving exam after 12 years, enabling them to pursue university education, lower-track schools present different school leaving exams and typically equip students for vocational paths or applied studies.

*Time of awakening & time since awakening.* Time of awakening and time since awakening were included in the models for sAA and cortisol to account for their diurnal rhythm over the measurement day. The time variables were included as hours, with time since awakening equaling 0 for the morning measurement.

### Statistical analyses

Statistical analyses were performed using R Statistical Software v4.2.3^84^. All biomarkers were found to exhibit positive skewness and were transformed by natural logarithm to normalize their distributions. Descriptive statistics for the analyzed variables were calculated. To investigate the pairwise relationships of the variables of interest, bivariate Pearson correlations were calculated, along with significance tests for the obtained correlations.

To investigate the moderating effects of the support variables on the relationships between the stress variables and the biomarker levels, moderation analyses were performed for all combinations of perceived stress and support variables for each biomarker type. Analyses were performed using linear models, with gender and study track included as covariates in all models. Models were built stepwise including: (a) covariates, (b) covariates and moderating variables and (c) covariates, moderating variables and their interaction. To investigate model fits, *R*^2^, Akaike Information Criterion (AIC) and Bayesian Information Criterion (BIC) values were calculated.

Since the students provided repeated measurements of cortisol and sAA during the measurement day, this necessitated accounting for within-subject effects for these analyses. First, due to both cortisol and sAA levels following known diurnal rhythms^36,85^, the time of awakening and time since awakening were included as covariates. Second, the inclusion of repeated observations from the subjects violates the assumption of independence of observations, leading to biased standard error estimates. To account for this, the standard errors of coefficient estimates were evaluated using cluster robust standard errors using the sandwich^86,87^ and lmtest^88^ packages. The cluster robust standard errors multiply residuals within each cluster, inflating the standard error estimates to the degree that the clustering is informative^89^.

Variables included in the analysis were checked for missing and outlier values. The students age was missing in 21 out of 83 cases, which is why it was not included as a covariate. One observation was excluded from sAA analysis due to undetectable values (< 1 μkatal/l) and two more due to improbably high values (> 10,000 μkatal/l). Two missing values were found in cortisol measurements, three in telomere lengths and one each in the time of awakening and time since awakening covariates. Eight instances of incomplete data were found in questionnaire items. Missing values in the biomarkers and covariates were handled by listwise deletion, while questionnaire scales were calculated using available data, due to less than 2.5% of the data missing in each item. Separate data sets were formed for each biomarker type to retain maximum sample size for moderation analyses.

## Results

Sample statistics are depicted in appendix Table 1.

### Correlation analyses

The correlation analyses (see appendix Table 2) indicated a general pattern of negative correlations between the stress and support variables. Support and stress variables showed no consistent relationships between the biomarkers, with the exceptions of telomere length and stress variables, which exhibited an overall negative relationship.

### Moderation analyses

In the following, we report moderation analyses with interactions significant at the p < 0.05 level. Tables describing moderation analyses are included in appendix Tables 3 and 4.

**Figure 1:**
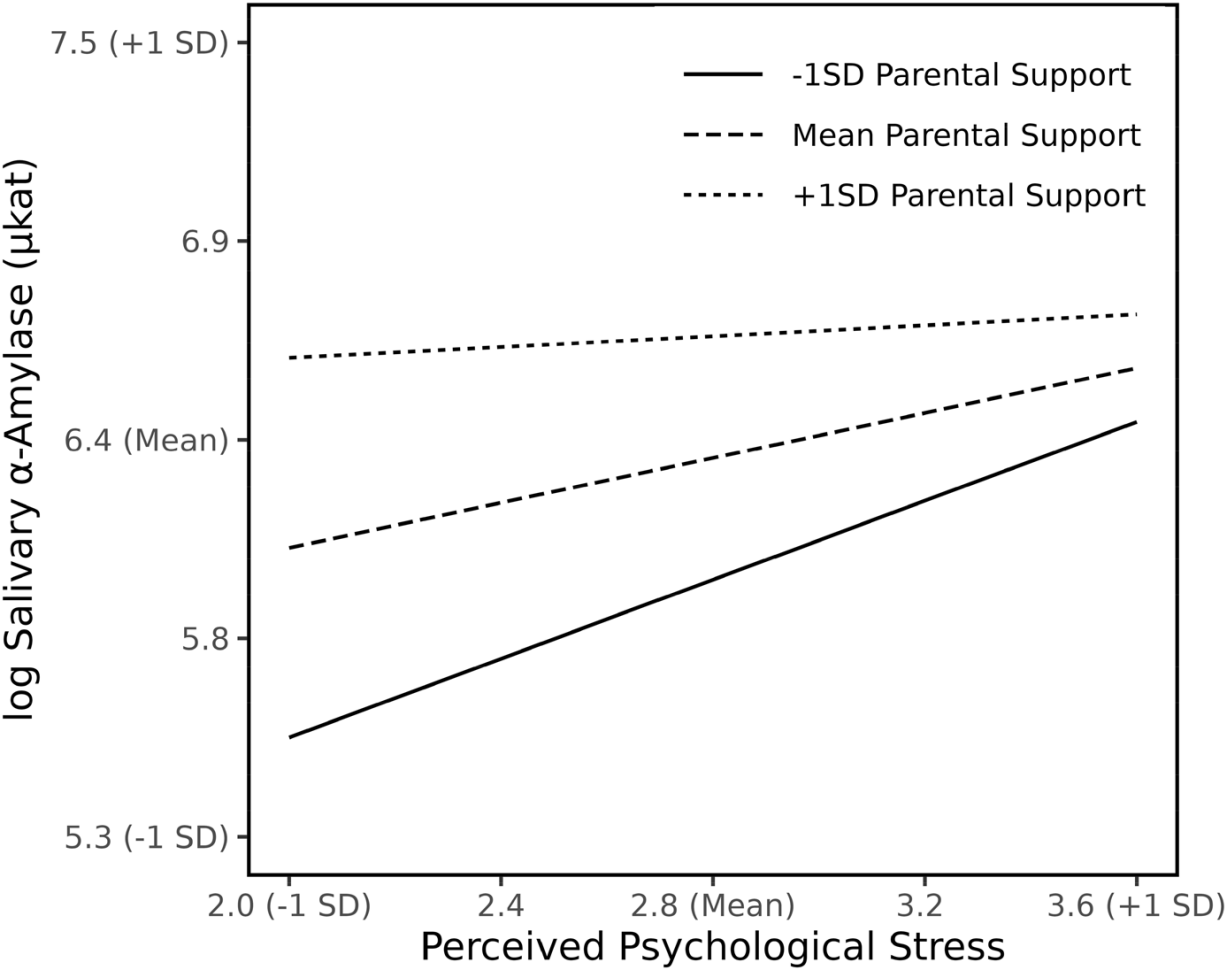
Interaction of Perceived Psychological Stress and Parental Support on sAA levels. Estimated simple slopes are plotted at mean and 1 SD above and below mean parental support levels. The level of sAA is expressed in logarithmized units.

*Perceived psychological stress and parental support* Both perceived psychological stress (*B* = 1.33, *crSE* = 0.38, *p* = 0.001) and parental support (*B* = 1.58, *crSE* = 0.43, *p* < 0.001) showed significant association with sAA, as well as a significant interaction related to sAA (*B* = -0.37, *crSE* = 0.12, *p* = 0.002) (appendix Table 3). The moderating effect is depicted in Fig. 1 and indicates that with increasing levels of psychological stress, students’ sAA levels increase. In detail, parental support changes significantly the variation rate of sAA related to psychological stress. The link between psychological stress and sAA increases to a lesser extent among students with high support compared to students who perceive lower support from their parents. For students perceiving lower parental support, the increase of sAA related to an increase of psychological stress is much steeper. In case of high psychological stress, the relationship between parental support and sAA weakens.

**Figure 2:**
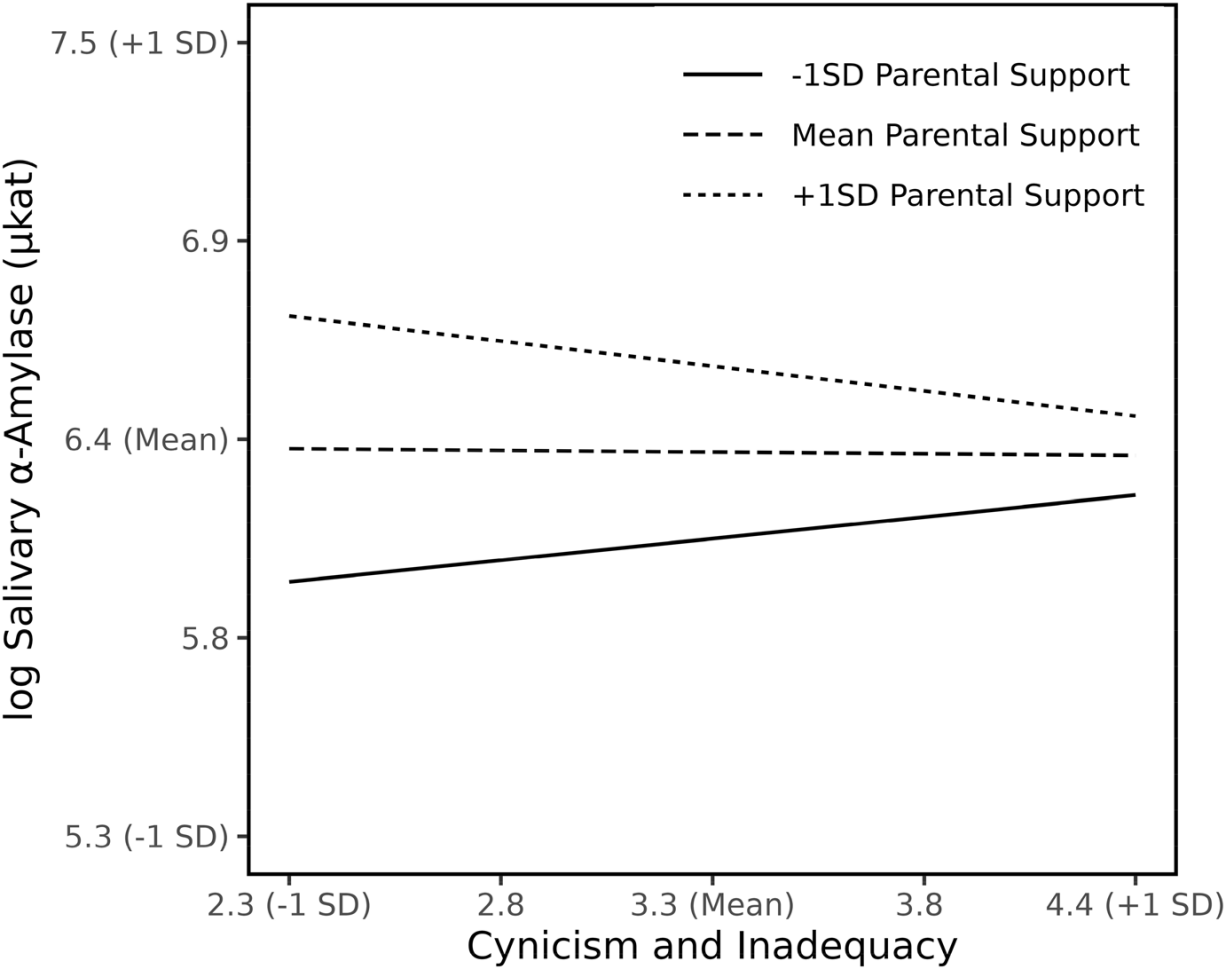
Interaction of Cynicism and Inadequacy with Parental Support on sAA levels. Estimated simple slopes are plotted at mean and 1 SD above and below mean parental support levels. The level of sAA is expressed in logarithmized units.

*Inadequacy/cynicism scale and parental support* The interaction of inadequacy/cynicism and parental support was significant (*B* = − 0.20, crSE = 0.10, *p* = 0.048), with both parental support (*B* = 1.06, crSE = 0.39, *p* = 0.007) and inadequacy/cynicism (*B* = 0.56, crSE = 0.32, *p* = 0.087) predicting larger sAA values (appendix Table 4). The moderating effect is shown in Fig. 2 and indicates that higher levels of parental support predicted overall higher levels of sAA related to low or average levels of inadequacy/cynicism. The effect of parental support changes the nature of the interplay of psychological stress and sAA. Students who experienced high parental support exhibited a decline of sAA related to an increase in inadequacy/cynicism whereas for students who experienced lower parental support this relationship shows a positive slope. For students who exhibit very high levels of inadequacy/cynicism the relationship with parental support diminishes in general.

## Discussion

The present study was designed to investigate how a) parental, b) teacher and c) peer support moderate the relationships between I) self-reported psychological stress and school burnout, namely II) exhaustion, and III) inadequacy/cynicism with biophysiological markers of stress and arousal including A) cortisol, B) alpha-amylase, C) oxidative stress, and D) telomere length. The aim of the study was to investigate protective factors of student’s immediate social environment that could serve as resources to overcome stressors, as theoretical frameworks, including the buffering hypotheses^12^ and the COR^13^ suggest that perceived social support mitigates feelings of stress and that social buffering reduces the activity of neurobiological systems associated with stress^60^.

According to hypothesis I, we expected a negative link between the social support variables and self-reported stress, exhaustion, and inadequacy/cynicism among students. This hypothesis was confirmed as students who perceived their parents, teachers, and peers as supportive reported lower levels of perceived psychological stress and inadequacy/cynicism. In addition, teacher support as well as peer support was linked to lower exhaustion among students. This finding is confirmed by various empirical studies that have investigated the protective role of parents^62,63^, teachers^29,73,74^, and peers^74,77^ when it comes to student’s perceived stress.

Following an exploratory approach, this study found that parental support functioned as buffer within the relationship between perceived psychological stress and sAA. In detail, it was found that the more time students spent with their parents, e.g., playing board games, having dinner together and doing the household, the higher their sAA was when their psychological stress was low or at an average level. In this case, sAA may reflect positive emotional arousal as students actively engage in activities together with their parents on a regular basis. The intermediate model in appendix Table 3 also indicated that as psychological stress increases among students, sAA levels also increase, reflecting heightened negative emotional arousal linked to psychological stress. As Adam et al.^37^ pointed out, sAA serves as a metric for assessing overall arousal levels, which can be triggered by either positive or negative emotions. Therefore, sAA is not exclusively associated with negative emotions or stress.

Interestingly, as psychological stress escalates among students, those who regularly spend time with their parents demonstrate minimal fluctuations in their sAA levels, whereas students who have limited interaction with their parents experience a sharp increase in sAA levels. Hence, common activities within the child-parent relationship buffer against increased stress related to sAA.

Parental support also acts as a protective buffer when the level of inadequacy and cynicism towards school rises in relation to sAA. Students who report an increasing sense of inadequacy at school and view school cynically tend to experience a decrease in sAA levels if they regularly spend time with their parents. Conversely, students who have limited interaction with their parents show a notable rise in sAA levels when cynicism and feelings of inadequacy at school increase. Again, parents function as buffer when it comes to students experiencing inadequacy and cynicism towards school as sAA levels are decreasing.

This is the first study to investigate parental support, measured by the time students spend with their parents, as moderator in relation to sAA and increasing levels of stress as well as inadequacy and cynicism towards school. The findings indicate that the time spent together with their parents protects students from experiencing high physiological arousal related to increasing stress levels and feelings of inadequacy and cynicism towards school. Hence, to ensure a healthy development of students, it is recommended for parents to make time for their children. This study highlights that the time spent in child-parent relationships can be an integral part of their daily life and routine, often involving ordinary and non-entertaining activities. Until now, research relying on self-report data has primarily connected the amount of time parents spend with their children to children’s well-being and behavioral issues but has not yet tested the moderation of parental-child time linked to stress^66–68^. However, there are various studies indicating that parental support plays an essential role for students’ stress, test anxiety, and whether students enjoy schools^62,63,90^.

Interestingly, no significant interaction effects with teacher support, peer support, self-reported variables and biophysiological markers was detected, which may possibly be due to the instruments used here, which surveyed peer support in a very general way. It is possible that support by close friends or peer network constructs could rather show a significant effect, as it has already been found in other studies^75,76^. Future studies should therefore test support by friends and social networks as a possible buffer. Another plausible interpretation for the lack of statistically significant interaction terms concerning peer support could also be attributed to the fact that stressed students may have a higher tendency to seek advice from adult professionals in comparison to their peers^91^ and the fear of social stigmatization and embarrassment may hold them back to address their need for help towards their peers^92^.

In sum, the results of the current study indicate that social support is related differently to students’ biophysiological markers, which are related to stress and arousal. Although theoretical models, such as the buffering hypothesis and COR propose that social support functions as resource in the face of stress, the current results suggest a more nuanced picture of social support perceived by students in relation to different stress contexts and biophysiological processes. Certainly, the connection between the variables of interest explored within intricate moderation models is complex. The variety of biophysiological stress and arousal markers as well as investigating different self-reported stress variables and sources of support allows us to draw a complex and complementary picture of how support relates to students’ self-reported stress and biophysiological markers.

In the quest to derive potential implications for stress prevention and intervention, it becomes imperative to accurately pinpoint the specific form of perceived stress experienced by individual students. This precision is essential for tailoring targeted support from both teachers and parents effectively.

### Strength, limitations, future directions

The current study is one of the few within the educational context that (a) combined several self-reported stress variables with a variety of biophysiological data, including hormones, enzymes, cell damage, and DNA of a healthy student cohort in Germany, (b) testing social support from parents, teachers and peers related to self-reported stress and biophysiological data.

Like any research endeavor, it is important to take into account certain constraints when interpreting the findings. Due to the exploratory design and small sample size, which impose certain limitations on statistical power, the results must be interpreted with caution. Replication studies with a larger sample are necessary to verify or falsify these first steps of basic research. The usage of manifest variables, though facilitating analysis, reduces variability. The alternative, latent structuring equation modeling, was not feasible due to the relatively small sample size. Furthermore, the relatively low reliability of the teacher support scale needs to be considered. However, following Kopp and Lois^93^, Cronbach’s alpha values above 0.50 are acceptable. Finally, the cross-sectional design of the study does not allow to detect any causal ordering of the variables. Accordingly, studies with a longitudinal design, which also measure biophysiological markers at several measurement points and follow students over a longer period to test potential changes in students’ subjective and biophysiological arousal, as well as changes in their perceived social support are necessary to detect potential causal relationships as well as developmental processes from childhood to adolescence. Overall, however, the present study lays an important foundation for understanding the role of social support in the interplay of different biophysiological markers and subjective stress variables in healthy secondary school students.

### Supplementary Information

Supplementary Information.

## Data Availability

Data will be made available on serious request. Please contact the corresponding author of this study.
