# Supplementary material for: Support from parents, teachers, and peers and the moderation of subjective and objective stress of secondary school student
Source: Sci Rep. 2024 Jan 12;14:1161. doi: 10.1038/s41598-024-51802-4 (PMC10786930; doi:10.1038/s41598-024-51802-4)
Supplement: Supplementary file 1 — Supplementary Information. [file 41598_2024_51802_MOESM1_ESM.docx]

**Appendix**

Appendix A. Analyzes of biophysiological stress markers

*Salivary Alpha-Amylase.* SAA from the morning and noon saliva samples were analyzed in duplicate using the AMY method on the Dimension Vista® system (Siemens Healthcare Diagnostics Inc, USA). The duplicate values of sAA were found to be highly reliable (*cor_morning_* = .984; *cor_noon_* = .995) and were averaged together for further analysis. SAA is reported in μkatal/l and can be interpreted as a measure of sympathetic nervous system activation via the SAM axis. In healthy individuals elevated levels of sAA are associated with increased levels of emotional arousal.

*Cortisol.* Salivary cortisol from the morning and noon samples were measured using the IDS-iSYS Multi-Discipline Automated System (Immunodiagnostic Systems Limited, United Kingdom), reported in nmol/l. Cortisol quantifies the systemic changes within the HPA axis, while elevated cortisol levels signify notable HPA axis activation due to stressors experienced by an individual. Salivary cortisol levels start high in the morning, peak within the first hour after awakening and then smoothly reduce during the day, while sAA exhibits approximately the opposite pattern (Miller et al., 2016; Nater et al., 2007).

*Oxidative stress.* An immunoassay kit, specifically the OxiSelect™ 8-iso-Prostaglandin F2a ELISA Kit from Cell Biolabs, Inc., was utilized to analyze a volume of 55 µl of urine. The immunoassay process and measurement adhered to the guidelines provided by the manufacturer. A Tecan Infinite 200 PRO instrument with a 450 nm filter was employed for photometric measurement. Using the obtained absorbance measurements, we determined the concentration of 8-iso-prostaglandin F2a present in the samples. Elevated oxidative stress levels arise due to the harmful impact of reactive oxygen species, signifying an unevenness between the generation and buildup of such species. This disparity can be prompted by a range of stress-inducing factors.

*Telomere length.* The saliva telomere samples were processed by using the prepIT•L2P | PT-L2P kit (DNA Genotek®, Steinbrenner Laborsysteme GmbH, Wiesenbach, Germany) following the manufacturer's instructions. The quality and concentration of the extracted DNA were assessed using a NanoDrop spectrophotometer (Isogen Life Science, Belgium). Units of telomere length are expressed in kilobases (kb). For more details on the analyses see xxxx (blinded for review).

Table 1. *Descriptive statistics for stress biomarkers; sAA, cortisol, oxidative stress, telomere length; psychological stress questionnaires: psychological stress, exhaustion, cynicism and inadequacy; support variables: parental support, teacher support, peer support.*

| Predictors | *N* | *M* | *SD* | *range* | *skewness* | *kurtosis* |
| --- | --- | --- | --- | --- | --- | --- |
| **Biomarkers** |  |  |  |  |  |  |
| Salivary α-Amylase (Morning) | 80 | 5.93 | 1.00 | 3.00 – 7.84 | -0.72 | 0.58 |
| Salivary α-Amylase (Midday) | 81 | 6.86 | 1.01 | 3.16 – 8.86 | -0.87 | 1.61 |
| Salivary Cortisol (Morning) | 82 | 2.60 | 0.67 | 0.52 – 4.03 | -0.18 | 0.03 |
| Salivary Cortisol (Midday) | 82 | 2.41 | 0.74 | 0.49 – 4.42 | 0.33 | 0.20 |
| Oxidative Stress | 83 | 7.09 | 0.61 | 5.30 – 8.41 | -0.51 | 0.37 |
| Telomere Length | 80 | 2.47 | 0.19 | 2.13 – 3.03 | 0.31 | -0.41 |
| **Covariates** |  |  |  |  |  |  |
| Time of awakening | 82 | 7.99 | 1.22 | 4.67 – 10.00 | -0.53 | -0.19 |
| Time after awakening | 81 | 2.89 | 1.13 | 1.50 – 5.83 | 1.08 | 0.29 |
| **Stress variables** |  |  |  |  |  |  |
| Psychological stress | 83 | 2.77 | 0.84 | 1.00 – 4.60 | 0.26 | -0.98 |
| Exhaustion | 83 | 2.66 | 1.14 | 1.00 – 5.50 | 0.48 | -0.56 |
| Cynicism and Inadequacy | 83 | 3.32 | 1.04 | 1.00 – 5.40 | -0.16 | -0.63 |
| **Support variables** |  |  |  |  |  |  |
| Parental Support | 83 | 2.79 | 0.62 | 1.60 – 4.00 | 0.08 | -0.84 |
| Teacher Support | 83 | 3.68 | 0.57 | 2.50 – 5.00 | -0.05 | -0.48 |
| Peer Support | 83 | 3.85 | 0.74 | 2.00 – 5.00 | -0.76 | -0.02 |

*Note: Values for the biomarkers are in units transformed by natural logarithm.*

Table 2. *Bivariate Pearson’s product-moment correlations for biomarkers, stress questionnaires and support-related variables.*

|  | 1. | 2. | 3. | 4. | 5. | 6. | 7. | 8. | 9. | 10. | 11. | 12. |
| --- | --- | --- | --- | --- | --- | --- | --- | --- | --- | --- | --- | --- |
| 1. Salivary α-Amylase (Morning) | - | **0.57***** | 0.26 | 0.12 | 0.18 | 0.07 | 0.26 | 0.07 | -0.02 | 0.16 | -0.01 | -0.08 |
| 2. Salivary α-Amylase (Noon) | **0.57***** | - | 0.02 | 0.00 | 0.10 | -0.05 | 0.13 | 0.05 | -0.11 | 0.22 | 0.11 | -0.01 |
| 3. Salivary Cortisol (Morning) | **0.26*** | 0.02 | - | **0.46**** | -0.02 | 0.35 | 0.01 | -0.08 | -0.06 | -0.07 | 0.02 | 0.05 |
| 4. Salivary Cortisol (Noon) | 0.12 | 0.00 | **0.46***** | - | 0.20 | 0.35 | -0.11 | -0.10 | -0.15 | 0.06 | 0.07 | 0.07 |
| 5. Oxidative Stress | 0.18 | 0.10 | -0.02 | 0.20† | - | 0.25 | 0.03 | -0.01 | -0.10 | -0.03 | 0.01 | 0.04 |
| 6. Telomere Length | 0.07 | -0.05 | **0.35**** | **0.35**** | **0.25*** | - | -0.22 | -0.28 | -0.23 | 0.07 | 0.10 | 0.10 |
| 7. Psychological Stress | **0.26*** | 0.13 | 0.01 | -0.11 | 0.03 | **-0.22*** | - | **0.71***** | **0.63***** | -0.24 | **-0.37*** | **-0.43**** |
| 8. Exhaustion | 0.07 | 0.05 | -0.08 | -0.10 | -0.01 | **-0.28*** | **0.71***** | - | **0.57***** | -0.18 | -0.32 | -0.35† |
| 9. Cynicism and Inadequacy | -0.02 | -0.11 | -0.06 | -0.15 | -0.10 | **-0.23*** | **0.63***** | **0.57***** | - | -0.28 | **-0.46***** | -0.30 |
| 10. Parental Support | 0.16 | **0.22*** | -0.07 | 0.06 | -0.03 | 0.07 | **-0.24*** | -0.18 | **-0.28*** | - | 0.17 | 0.24 |
| 11. Teacher Support | -0.01 | 0.11 | 0.02 | 0.07 | 0.01 | 0.10 | **-0.37***** | **-0.32**** | **-0.46***** | 0.17 | - | **0.39*** |
| 12. Peer Support | -0.08 | -0.01 | 0.05 | 0.07 | 0.04 | 0.10 | **-0.43***** | **-0.35**** | **-0.30**** | **0.24*** | **0.39***** | - |

*Note*: Significance levels are reported at †p < .1, *p < .05, **p < .01, ***p < .001. Correlations with significance of p < .05 are bolded. Bonferroni correction has been applied for all correlations in the upper triangle. Biomarkers, α-Amylase, cortisol, oxidative stress and telomere length are included as logarithmized values.

Table 3. *Moderation analysis – Perceived psychological stress, parental support and levels of salivary α-amylase.*

|  | | | | | | |
| --- | --- | --- | --- | --- | --- | --- |
|  | **Dependent Variable: log Salivary α-Amylase** | | | | | |
|  |  | | | | | |
| **Predictors** | B *(crSE)* | | | *P* | | |
|  | | | | | | |
| **Control Variables** |  |  |  |  |  |  |
| Time of awakening (hours) | 0.05 (0.07) | 0.05 (0.07) | 0.06 (0.06) | .491 | .427 | .315 |
| Time after awakening (hours) | 0.31*** (0.03) | 0.30*** (0.03) | 0.30*** (0.03) | .000 | .000 | .000 |
| Gender (male) | -0.11 (0.20) | -0.04 (0.19) | -0.02 (0.20) | .606 | .827 | .931 |
| School type (high-tracking) | 0.05 (0.21) | -0.08 (0.20) | -0.09 (0.20) | .794 | .689 | .649 |
| **Predictors** |  |  |  |  |  |  |
| Perceived psychological stress |  | 0.26* (0.12) | 1.33*** (0.38) |  | .027 | .001 |
| Parental support |  | 0.47*** (0.13) | 1.58*** (0.43) |  | .001 | .000 |
| **Interaction Term** |  |  |  |  |  |  |
| Perceived psychological stress x Parental support |  |  | -0.37** (0.12) |  |  | .002 |
|  | | | | | | |
| **R2** | .21 | .29 | .32 |  |  |  |
| **ΔR²** |  | .08 | .04 |  |  |  |
| **AIC** | 453.17 | 441.32 | 435.19 |  |  |  |
| **BIC** | 471.54 | 465.82 | 462.75 |  |  |  |
|  | | | | | | |
| Note. Reported are unstandardized regression coefficient estimates (B) from ordinary least squares models, cluster robust standard errors (crSE) and p-values (P). Significance levels are reported at †p < .1; *p < .05; **p < .01; ***p < .001. Waking up time and time delay after waking up are measured in hours, with time delay after waking up coded as 0 for the morning measurement. Gender; 0 = girls, 1 = boys, School type; 0 = low-track school, 1 = high-track school. R² = Coefficient of determination, ΔR² = Change in R² between the models, AIC = Akaike Information Criterion, BIC = Bayesian Information Criterion. | | | | | | |

Table 4. *Moderation analysis – Inadequacy/cynicism, parental support and levels of salivary α-amylase.*

|  | | | | | | |
| --- | --- | --- | --- | --- | --- | --- |
|  | **Dependent Variable: log Salivary α-Amylase** | | | | | |
|  |  | | | | | |
| **Predictors** | B *(crSE)* | | | *P* | | |
|  | | | | | | |
| **Control Variables** |  |  |  |  |  |  |
| Time of awakening (hours) | 0.05 (0.07) | 0.05 (0.07) | 0.06 (0.07) | .491 | .467 | .449 |
| Time after awakening (hours) | 0.31*** (0.03) | 0.31*** (0.03) | 0.31*** (0.03) | .000 | .000 | .000 |
| Gender (male) | -0.11 (0.20) | -0.13 (0.21) | -0.12 (0.21) | .606 | .529 | .571 |
| School type (high-tracking) | 0.05 (0.21) | -0.04 (0.20) | -0.05 (0.20) | .794 | .847 | .800 |
| **Predictors** |  |  |  |  |  |  |
| Inadequacy/cynicism |  | -0.02 (0.08) | 0.56† (0.32) |  | .801 | .087 |
| Parental support |  | 0.36* (0.14) | 1.06** (0.39) |  | .010 | .007 |
| **Interaction Term** |  |  |  |  |  |  |
| Inadequacy/cynicism x Parental support |  |  | -0.20* (0.10) |  |  | .048 |
|  | | | | | | |
| **R2** | .21 | .25 | .27 |  |  |  |
| **ΔR²** |  | .04 | .02 |  |  |  |
| **AIC** | 453.17 | 448.74 | 447.44 |  |  |  |
| **BIC** | 471.54 | 473.24 | 475.01 |  |  |  |
|  | | | | | | |
| Note. Reported are unstandardized regression coefficient estimates (B) from ordinary least squares models, cluster robust standard errors (crSE) and p-values (P). Significance levels are reported at †p < .1; *p < .05; **p < .01; ***p < .001. Waking up time and time delay after waking up are measured in hours, with time delay after waking up coded as 0 for the morning measurement. Gender; 0 = girls, 1 = boys, School type; 0 = low-track school, 1 = high-track school, inadequacy/cynicism = factor combining cynicism and inadequacy. R² = Coefficient of determination, ΔR² = Change in R² between the models, AIC = Akaike Information Criterion, BIC = Bayesian Information Criterion. | | | | | | |
